# Supplementary material for: A plasmid network from the gut microbiome of semi-isolated human groups reveals unique and shared metabolic and virulence traits
Source: Sci Rep. 2022 Jul 15;12:12102. doi: 10.1038/s41598-022-16392-z (PMC9287393; doi:10.1038/s41598-022-16392-z)
Supplement: Supplementary file 1 — Supplementary Information 1. [file 41598_2022_16392_MOESM1_ESM.pdf]

Supplementary Note 1. Diet and lifestyle of the semi-isolated human groups studied

Yanomami

The Yanomami, a hunter-gatherer group that occasionally cultivates plantains and cassava, inhabits a huge region in Amazon, encompassing the Brazil and Venezuela border. Their villages can be located at sea level as well as on high mountains, and therefore, their diets mainly depend on the resources of the niche inhabited and seasons. In general, they rely on the variety of foods seasonally available in the rainforest, which includes snakes, wild pigs, monkeys, deer, and jaguars, but also insects, larvae, fish, crabs, wild honey, roots, and various fruits and vegetables. Their diet is low in fat and salt and high in fiber. The drinking water is collected directly from unprotected wells and river streams [1].

Matses

The Matses, a remote hunter-gatherer group from the Peruvian Amazon, has been geographically, historically, and socially, isolated until recently. They live at an elevation of 150 m above sea level. The Matses diet primarily consists of varieties of manioc and plantain/banana, which are rudimentarily cultivated around their settlement. But their diet also includes other crops like sweet potato, maize, rice, cacao, and various fruits, and any other crop brought from outside the community. Fish is their primary protein source, but they sporadically consume meat from mammals (such as monkeys, sloths, and peccary), reptiles, and birds. And they also have turtle and alligator eggs with an irregular frequency [2].

Tunapuco

The Tunapuco is a traditional agricultural community from the Andean highlands, situated in the central Andes, at an elevation between 2,500 and 3,100 m above sea level. Their diet is based on local agriculture and homegrown small animals. The Tunapuco staple food includes potatoes, Andean tubers, artisan bread, hominy, rice, garlic, carrots, cabbages, and faba beans. But depending on the season, they also have access to various fruits, such as apples, oranges, melon pears, and bananas. Their meat sources are mammals (such as guinea pigs, beef, pork, and sheep), birds, and fish. The intake of chicken eggs and dairy, such as natural fresh milk, and fresh white cheese is limited. Store-bought products are also part of their weekly diet, including oatmeal, pasta, sugar, black pepper, cumin, canned condensed milk, and cooking oil [2].

Hadza

The Hadza maintain a hunter-gatherer subsistence lifestyle, relying on the wild foods they find in their region, situated in the Rift Valley ecosystem around the shores of Lake Eyasi in northwestern Tanzania. The Hadza gut microbiome data analyzed here comes from fecal samples collected during the rainy season. During the rainy season, their diet mostly consists of plant foods (such as species of tuber, small and large game, honey from stinging and stingless bees, leafy green foliage, baobab fruit, and one species of berry), whereas during the dry season, more meat is consumed. Tough insoluble fibers are largely and frequently consumed by the Hadza. They chew on the fibers for a short period of time (one to three minutes), wadge it to remove all moisture and pulp, and then expectorate as a "quid" [3,4].

References:

1. Conteville LC, Oliveira-Ferreira J, Vicente ACP. Gut Microbiome Biomarkers and Functional Diversity within an Amazonian Semi-Nomadic Hunter-Gatherer Group. Front Microbiol. 2019 Jul 30;10:1743.

2. Obregon-Tito AJ, Tito RY, Metcalf J, Sankaranarayanan K, Clemente JC, Ursell LK, Zech Xu Z, Van Treuren W, Knight R, Gaffney PM, Spicer P, Lawson P, Marin-Reyes L, Trujillo-Villarroel O, Foster M, Guija-Poma E, Troncoso-Corzo L, Warinner C, Ozga AT, Lewis CM. Subsistence strategies in traditional societies distinguish gut microbiomes. Nat Commun. 2015 Mar 25;6:6505.

3. Schnorr SL, Candela M, Rampelli S, Centanni M, Consolandi C, Basaglia G, Turroni S, Biagi E, Peano C, Severgnini M, Fiori J, Gotti R, De Bellis G, Luiselli D, Brigidi P, Mabulla A, Marlowe F, Henry AG, Crittenden AN. Gut microbiome of the Hadza hunter-gatherers. Nat Commun. 2014 Apr 15;5:3654.

4. Rampelli S, Schnorr SL, Consolandi C, Turroni S, Severgnini M, Peano C, Brigidi P, Crittenden AN, Henry AG, Candela M. Metagenome Sequencing of the Hadza Hunter-Gatherer Gut Microbiota. Curr Biol. 2015 Jun 29;25(13):1682-93
